# Editorial: New insights in sepsis pathogenesis and renal dysfunction: Immune mechanisms and novel management strategies

**DOI:** 10.3389/fimmu.2023.1176620

**Published:** 2023-03-08

**Authors:** Alessandra Stasi, Patrick M. Honore

**Affiliations:** ^1^ Nephrology, Dialysis and Transplantation Unit, Department of Precision and Regenerative Medicine and Ionian Area, University of Bari “Aldo Moro”, Bari, Italy; ^2^ Intensive Care Unit (ICU) Department, Université Catholique de Louvain (UCL) Louvain Medical School, Centre Hospitalier Universitaire (CHU) UCL Godinne Namur, Yvoir, Belgium

**Keywords:** sepsis, molecular mechanism, omics, animal model, therapeutic strategy, immunology & infectious diseases, acute kidney injury

Sepsis is one of the main causes of admission in Intensive Care Units (ICU) with an estimated 13.7 million of deaths globally for 2019 (1). Sepsis is a complex disease characterized by a systemic inflammatory response that arises during infection. Gram negative bacteria are considered the common pathogens involved in sepsis disease (2). The host response includes pro-inflammatory and anti-inflammatory mechanisms that are necessary for pathogen clearance, but in this context, they could become impaired and lead to systemic organ damage (3). There is currently no cure for this disease which affects a broad set of population starting from infants to elderly people. Nevertheless, there were some treatment attempts targeting either the endotoxin inactivation/elimination or some cytokines which are supposedly keys in the detrimental cytokine storm induced by either bacterial or viral (like the recent sars-Cov-2) infections and which is the hallmark of sepsis.

Therefore, more efforts have to be addressed to better understand the pathogenesis of this disease and discover novel targeting candidates for innovative therapeutic approaches.

We, as co-guest editors, are pleased to present the first volume of the Research Topic collection “*New Insights in Sepsis Pathogenesis and Renal Dysfunction: Immune Mechanisms and Novel Management Strategies*” in Frontiers in Immunology. This volume aimed to cover the multiple mechanisms associated with the dysfunctional immune response and life-threatening organ dysfunction that commonly includes acute kidney injury (AKI). The first study submitted and accepted for the current Research Topic details the controversial role and heterogeneity of Treg in sepsis, defined as angels or demons and possible target for new therapeutic approaches (Gao et al.).

Several original research articles accepted in this collection report new insights into the molecular mechanisms that exacerbate the renal injury during sepsis. The study by Zhang et al. presented a systematic single cell transcriptomic atlas of AKI by dissecting the key cellular player and molecular mechanisms associated with AKI onset. In particular, the authors identified pre-injured proximal tubule cells (PTC) subtypes and their pro-inflammatory and pro-fibrotic signature.

Interestingly, Chen et al. clarified the exact role of STAT3 activation in sepsis-induced AKI, introducing a new intriguing mechanism that could display remarkable protective effect and could offer a new therapeutic target to prevent acute tubular damage.

In line with recent literature, animal models such as murine and rat models are valuable and extensively used for the investigation of pathogenesis and verification of potential treatment in sepsis associated immunosuppression. Wang et al. summarized the current knowledge of murine models to investigate sepsis disease, highlighting their advantages and limitations, proposing new directions in refining murine sepsis models to increase the clinical relevance and optimizing their value of meeting research and translational demands. In addition, Cao et al. successfully developed a standardized model of urinary sepsis in rats that could have a great relevance in the translational research.

Actually, the research is strongly involved in finding new injury or stress markers that could guide clinicians to recognize the first signs of renal injury and to avoid disease progression. The early diagnosis of AKI in sepsis, together with the use of antibiotics and appropriate fluid therapy may provide an optimal strategy to counteract renal injury progression and worsen outcome associated with poor survival (4). In this context, Qiao and Cui introduced the application of multiple “omics” techniques to discover new mechanisms in the pathophysiology of SI-AKI and to find more effective biomarkers that could accelerate diagnosis and provide the possibility of individualized treatment. Interestingly, Xiao et al. presented several statistical methods, such as competing risks models and double robust estimation, to evaluate the association between the ratio of neutrophils to lymphocytes and platelets (N/LP) with the incidence of S-AKI and severe AKI in sepsis patients. Then, enhanced monitoring of UO and N/LP would be more helpful in guiding clinical decisions about S-AKI. Finally, Wang et al. established a risk prediction model to assess the probability of developing AKI in Hemophagocytic lymphohistiocytosis (HLH) patients. The occurrence of this clinical syndrome, caused by an uncontrolled immune response, as in sepsis, is associated with a higher percentage of multiorgan failure, including kidney failure and death.

Sex-stratified medicine is an important aspect of precision medicine. Previous studies are mainly about the effect of sex on mortality of SI-AKI patients, and data on the association between sex and the incidence, risk factors and clinical impact of SI-AKI are not exhaustive. In a large retrospective study, Peng et al. provided evidences that sex-related effects may play a minor role in the incidence and the clinical course of SI-AKI, and the association between sex and the clinical management was insignificant in the full-adjusted model.

Current treatment protocols for septic patients are based upon source control,hemodynamic resuscitation, supportive therapy and adequate antibiotic therapy. However, in most critically ill patients, these measures are not enough to prevent sepsis-related organ dysfunction and the onset of AKI. In the study by Anter et al. the authors demonstrated for the first time the effect of menthol on an experimental model of sepsis (cecal ligation and puncture model). They found that menthol improved the survival of rats after induction of sepsis and protected the lung and kidneys, showing an ameliorative effect against enhanced oxidative stress induced by sepsis. Finally, the authors explored the pathways mediating the protective effect of menthol, and they introduced anti-proliferating cell nuclear antigen (PCNA) as a new target for sepsis therapy.

Since other investigations have to be carried out to identify other potential multi-protective mediators that could regulate a broad spectrum of events that occurred in sepsis pathogenesis, we are pleased to launch the volume II of this Research Topic.

## Author contributions

AS compiled first and final version of the manuscript. PMH revised the manuscript. All authors contributed to the article and approved the submitted version.
